# Attitudes and Intentions of US Veterans Regarding COVID-19 Vaccination

**DOI:** 10.1001/jamanetworkopen.2021.32548

**Published:** 2021-11-03

**Authors:** Guneet K. Jasuja, Mark Meterko, Ledjona D. Bradshaw, Richard Carbonaro, Marla L. Clayman, Lara LoBrutto, Danielle Miano, Elizabeth M. Maguire, Amanda M. Midboe, Steven M. Asch, Allen L. Gifford, D. Keith McInnes, A. Rani Elwy

**Affiliations:** 1 Bridge Quality Enhancement Research Initiative Program, Center for Healthcare Organization and Implementation Research, VA Bedford Healthcare System, Bedford, Massachusetts; 2 Section of General Internal Medicine, Boston University School of Medicine, Boston, Massachusetts; 3 Office of Analytics and Performance Integration, Survey of Healthcare Experiences of Patients, Veterans Health Administration, Washington, District of Columbia; 4 Department of Health Law, Policy and Management, Boston University School of Public Health, Boston, Massachusetts; 5 Bridge Quality Enhancement Research Initiative Program, Center for Healthcare Organization and Implementation Research, VA Boston Healthcare System, Jamaica Plain, Massachusetts; 6 Bridge Quality Enhancement Research Initiative Program, Center for Innovation to Implementation, VA Palo Alto Healthcare System, Menlo Park, California; 7 Center for Primary Care and Outcomes Research, Stanford University School of Medicine, Stanford, California; 8 Division of Primary Care and Population Health, Stanford University School of Medicine, Stanford, California; 9 Department of Psychiatry and Human Behavior, Alpert Medical School, Brown University, Providence, Rhode Island

## Abstract

**Importance:**

Compared with the general population, veterans are at high risk for COVID-19 and have a complex relationship with the government. This potentially affects their attitudes toward receiving COVID-19 vaccines.

**Objective:**

To assess veterans’ attitudes toward and intentions to receive COVID-19 vaccines.

**Design, Setting, and Participants:**

This cross-sectional web-based survey study used data from the Department of Veterans Affairs (VA) Survey of Healthcare Experiences of Patients’ Veterans Insight Panel, fielded between March 12 and 28, 2021. Of 3420 veterans who were sent a link to complete a 58-item web-based survey, 1178 veterans (34%) completed the survey. Data were analyzed from April 1 to August 25, 2021.

**Exposures:**

Veterans eligible for COVID-19 vaccines.

**Main Outcomes and Measures:**

The outcomes of interest were veterans’ experiences with COVID-19, vaccination status and intention groups, reasons for receiving or not receiving a vaccine, self-reported health status, and trusted and preferred sources of information about COVID-19 vaccines. Reasons for not getting vaccinated were classified into categories of vaccine deliberation, dissent, distrust, indifference, skepticism, and policy and processes.

**Results:**

Among 1178 respondents, 974 (83%) were men, 130 (11%) were women, and 141 (12%) were transgender or nonbinary; 58 respondents (5%) were Black, 54 veterans (5%) were Hispanic or Latino, and 987 veterans (84%) were non-Hispanic White. The mean (SD) age of respondents was 66.7 (10.1) years. A total of 817 respondents (71%) self-reported being vaccinated against COVID-19. Of 339 respondents (29%) who were not vaccinated, those unsure of getting vaccinated were more likely to report fair or poor overall health (32 respondents [43%]) and mental health (33 respondents [44%]) than other nonvaccinated groups (overall health: range, 20%-32%; mental health: range, 18%-40%). Top reasons for not being vaccinated were skepticism (120 respondents [36%] were concerned about side effects; 65 respondents [20%] preferred using few medications; 63 respondents [19%] preferred gaining natural immunity), deliberation (74 respondents [22%] preferred to wait because vaccine is new), and distrust (61 respondents [18%] did not trust the health care system). Among respondents who were vaccinated, preventing oneself from getting sick (462 respondents [57%]) and contributing to the end of the COVID-19 pandemic (453 respondents [56%]) were top reasons for getting vaccinated. All veterans reported the VA as 1 of their top trusted sources of information. The proportion of respondents trusting their VA health care practitioner as a source of vaccine information was higher among those unsure about vaccination compared with those who indicated they would definitely not or probably not get vaccinated (18 respondents [26%] vs 15 respondents [15%]). There were no significant associations between vaccine intention groups and age (χ^2^_4_ = 5.90; *P* = .21) or gender (χ^2^_2_ = 3.99; *P* = .14).

**Conclusions and Relevance:**

These findings provide information needed to develop trusted messages used in conversations between VA health care practitioners and veterans addressing specific vaccine hesitancy reasons, as well as those in worse health. Conversations need to emphasize societal reasons for getting vaccinated and benefits to one’s own health.

## Key Points

**Question:**

What are veterans’ attitudes and intentions associated with COVID-19 vaccination?

**Findings:**

In this survey study of 1178 US veterans in March 2021, 71% of veterans reported being vaccinated against COVID-19. Fears about side effects and worry about the newness of vaccines were the primary reasons given for not getting vaccinated, reflecting vaccine skepticism and deliberation.

**Meaning:**

These findings suggest that targeting veterans’ concerns around the adverse effects and safety of COVID-19 vaccines through conversations with trusted Veterans Health Administration health care practitioners is key to increasing vaccine acceptance.

## Teaser

This survey study assesses veterans’ attitudes toward and intentions to receive COVID-19 vaccines.

## Introduction

COVID-19 is a leading cause of death in the United States.^1^ As of early October 2021, there have been 15 243 known deaths among veterans receiving care in the Veterans Health Administration (VHA) and 704 233 deaths in the United States attributed to COVID-19.^2,3^ Vaccines will end the pandemic only if enough people are willing to become fully vaccinated.^4^ Several cross-sectional surveys conducted among US adults have captured varying influences on attitudes and intentions associated with COVID-19 vaccine hesitancy, defined as the delay in acceptance or refusal of vaccination despite availability of vaccination services.^5^ Fears concerning COVID-19 vaccine safety and fast-tracked vaccine development are barriers to vaccine acceptance.^6,7^ Prior influenza vaccinations,^6,7,8^ trust in government,^7,9^ health care practitioner recommendations,^10,11^ high perceived likelihood of getting COVID-19, and perceived severity of illness^12^ are important facilitators for vaccine acceptance.

Veterans are a group who experience greater disease burden from COVID-19.^13,14^ As trust in government is an important factor associated with vaccine acceptance,^7^ vaccine hesitancy in veterans receiving care in the VHA, who are former soldiers and direct recipients of government care, requires examination, considering veterans have a complex relationship with the government compared with the general population.^9,15^ However, little is known about the attitudes and intentions of veterans regarding COVID-19 vaccination in the VHA, the largest integrated health care system in the US. The VHA has enough vaccine supply for any veteran who wishes to be vaccinated, and indeed, 3.6 million veterans have been vaccinated within the VHA.^2^

As an embedded quality improvement team within the VHA system,^16^ our task was to rapidly yet rigorously evaluate the implementation of COVID-19 vaccines. Thus, the goal of this study was to assess veterans’ attitudes and intentions regarding COVID-19 vaccination in the specific context of the VHA system to inform ongoing, systemwide communication efforts to increase the uptake of the vaccines.

## Methods

This survey study received an institutional review board exemption from the VA Bedford Healthcare System as a quality improvement project. As a quality improvement activity, participants do not provide written informed consent to participate in this or ongoing surveys, but participation is voluntary and does not have any effect on their VHA healthcare or benefits. We followed the American Association for Public Opinion Research (AAPOR) reporting guideline for survey studies.

### Study Sample

The sample for the study was drawn from the Department of Veterans Affairs (VA) Survey of Healthcare Experiences of Patients’ (SHEP) Veteran Insights Panel (VIP).^17^ SHEP, part of the VHA Office of Analytics and Performance Integration, provides information to facility managers about wide-ranging features of the veteran health care experience, including access, communication with health care practitioners, and coordination of care.^17^ The VIP is a standing, national online group of veterans who regularly use VA health care, created to enable veterans’ rapid feedback on VA programs and services on an as-needed basis. Details of the VIP have been reported elsewhere.^18^ A total of 3420 members of the VIP were invited via email to participate in a web-based survey fielded March 12 to 28, 2021. The VHA began vaccinating older veterans, those with chronic health conditions, and those in long-term care facilities on December 14, 2020.^2^ In January 2021, veterans’ access increased, with those 50 years and older eligible to receive vaccinations. Approximately 2.5 months elapsed between vaccine access and completion of this survey.

Of 3420 veterans sent a link to complete the survey, 1194 (35%) responded. All veterans contacted were eligible to participate in the study. Surveys from 16 veterans were found to be substantially incomplete and not included in any analyses. The remaining 1178 surveys represented a 34% response rate and constituted the final analytic database for this study.

### Survey Variables

The 58-item survey was developed in collaboration with SHEP and Ipsos Public Affairs, who manages the VIP. We incorporated COVID-19 questions from ongoing veteran surveys (F. Weaver, PhD, email, January 7, 2021; H. Gordon, MD, email, January 19, 2021)^19^ and by conducting a literature review for other survey items.^20,21,22,23,24^ Respondents self-reported their age, gender, race, ethnicity, overall health status, and mental health status using standard question items from SHEP and VA surveys.^17,25,26^ The final survey was pilot tested in a 10% random sample including 342 veterans in the VIP. Pilot data were checked for several quality features (eg, amount of missing data, appropriateness of responses given skip-pattern logic), and no problems were found.

The survey assessed veterans’ experience with and exposure to COVID-19, COVID-19 vaccination status and intentions of getting the vaccine, reasons for not being vaccinated among those not already vaccinated, reasons for getting vaccinated among those who already were or who intended to get vaccinated, trusted sources of information, and preferred sources of vaccine information. We mapped the reasons for not getting vaccinated to 1 of 5 types of hesitancy categories^27^: (1) vaccine deliberation: watchful waiting or need for more data on vaccine safety; (2) vaccine dissent: not in favor of vaccines in general; (3) vaccine distrust: owing to involvement of the government and/or historical and present-day inequities; (4) vaccine indifference: not concerned about COVID-19 and thus do not see the need for a vaccine; and (5) vaccine skepticism: fears of illness, unnatural substances, and elite conspiracy. We created a policy and process category, to capture logistical challenges veterans reported with accessing vaccines, and which indicated a nonhesitancy reason for not yet being vaccinated.^28^ Categories were assigned to veterans’ reported reasons for not getting vaccinated through a discussion and consensus process among 3 of us (G.K.J., M.M., and A.R.E.), yet it is recognized that these categories are not necessarily mutually exclusive.^28^ The full survey is available in the eAppendix in the Supplement.

### Statistical Analysis

Descriptive statistics were used to analyze frequency of exposure to COVID-19, COVID-19 vaccination status, vaccine hesitancy, common barriers to vaccination, effective messengers, and preferred presentations of vaccine information. Bivariate analyses tested for associations between vaccine intention groups (definitely will not, probably will not, not sure, probably will, definitely will), exposure to COVID-19, and demographic characteristics. We also explored how reasons for not getting a vaccine, trusted sources of information, and preferred modes of communication varied among those with different levels of vaccination intention. We performed all analyses using SAS statistical software version 9.4 (SAS Institute). *P* values were 2-sided, and statistical significance was set at .05. Data were analyzed from April 1 to August 25, 2021.

## Results

### Experience With COVID-19 and COVID-19 Vaccination

Of 1178 study respondents, 974 (83%) were men, 130 (11%) were women, and 11 (<1%) were transgender, nonbinary or other; 58 respondents (5%) were Black, 54 veterans (5%) were Hispanic or Latino, and 987 veterans (84%) were non-Hispanic White (Table 1), similar to the greater VA population.^29^ The mean (SD) age was 66.7 (10.1) years. A total of 123 respondents (10%) reported having had COVID-19 themselves. Most respondents (817 of 1156 respondents [71%] who answered questions about vaccination status) reported having already been vaccinated against COVID-19. Of those not yet vaccinated, 91 respondents (27%) said they definitely will not get vaccinated, 44 respondents (13%) reported probably will not, 76 respondents (22%) were unsure, 43 respondents (13%) said probably will, and 85 respondents (25%) indicated they definitely will get a COVID-19 vaccination.

**Table 1.** Demographics and Experience With COVID-19 and COVID-19 Vaccination

Characteristic	No. (%) (N = 1178)^a^
Gender	
Male	974 (83)
Female	130 (11)
Transgender man	0
Transgender woman	4 (<1)
Nonbinary	2 (<1)
Other	5 (<1)
Missing	63 (5)
Race and ethnicity	
Hispanic or Latino	54 (5)
American Indian or Alaska Native	10 (1)
Asian	5 (<1)
Black or African American	58 (5)
Multiracial	52 (4)
Native Hawaiian or other Pacific Islander	3 (<1)
White	987 (84)
Missing or unknown	63 (5)
Had COVID-19	
Yes	123 (10)
No	989 (84)
Not sure	59 (5)
Received a COVID-19 vaccine	817 (71)
Vaccine intention (among those who have not been vaccinated)	
Definitely will not	91 (27)
Probably will not	44 (13)
Not sure	76 (22)
Probably will	43 (13)
Definitely will	85 (25)

^a^ Respondents were not forced to answer each relevant survey question before continuing; therefore, the total number of respondents for each variable does not always equate to 1178.

### Reasons Why Veterans Had Not Yet Been Vaccinated Against COVID-19

Among 339 respondents not yet vaccinated, vaccine skepticism was the topmost reason for not getting vaccinated, with 120 respondents (36%) stating concerns about the adverse effects from COVID-19 vaccines, 65 respondents (20%) preferring to use as few medicines as possible, and 63 respondents (19%) seeking to gain natural immunity. Other reasons for not getting vaccinated ranged from vaccine deliberation (74 respondents [22%] were concerned about the newness of the COVID-19 vaccine); vaccine distrust (61 respondents [18%] reported a lack of trust in the health care system); and vaccine dissent (48 respondents [15%] did not believe in vaccines in general). Policy and process issues, such as not yet eligible (25 respondents [8%]) or lack of transportation (5 respondents [2%]), reflected some reasons for not being vaccinated, as opposed to vaccine hesitancy (Table 2).

**Table 2.** Reasons Rated as Highly Important for Why an Individual Has Not Yet Received a COVID-19 Vaccination by Vaccine Hesitancy Categories

Reason^a^	No. (%) (n = 339)	Vaccine hesitancy category
I am concerned about side effects from the vaccine.	120 (36)	Vaccine skepticism
The COVID[-19] vaccine is new, so I want to wait a while before deciding.	74 (22)	Vaccine deliberation
I prefer to use as few medicines as possible.	65 (20)	Vaccine skepticism
I prefer gaining natural immunity.	63 (19)	Vaccine skepticism
I do not trust the health care system to act in my best interests.	61 (18)	Vaccine distrust
I do not trust vaccines.	48 (15)	Vaccine dissent
I am eligible for a COVID-19 vaccine but have not yet been able to get an appointment.	44 (13)	Policy and process
I am worried it will alter my DNA.	40 (12)	Vaccine skepticism
I am not eligible for a COVID-19 vaccine at this time.	25 (8)	Policy and process
You need to get 2 shots about 1 mo apart for the vaccines to work.	25 (8)	Policy and process
I am allergic to vaccines.	23 (7)	Vaccine dissent
Getting a COVID-19 vaccine is too difficult.	23 (7)	Policy and process
It is against my religious or philosophical beliefs.	20 (6)	Vaccine dissent
I don’t know how to get a COVID-19 vaccine.	19 (6)	Policy and process
I am concerned about vaccine safety related to pregnancy and/or breastfeeding.	17 (5)	Vaccine skepticism
I’ve already had COVID-19 so I don’t believe that I need a COVID-19 vaccine.	13 (4)	Vaccine indifference
I do not like needles.	11 (3)	Vaccine skepticism
I don’t have transportation.	5 (2)	Policy and process

^a^ Respondents were provided the list of 18 reasons and asked to rate each as not important, slightly important, important, very important, or highly important and were also provided an option to provide their own reasons.

### Association Among Vaccine Intention Groups, Health Status, and Demographic Characteristics

As reported in the Figure, A, veterans who were not sure about vaccination described their overall health as fair or poor (32 respondents [43%]) compared with 11 respondents (28%) in the probably will get vaccinated, 24 respondents (32%) in the definitely will get vaccinated, 8 respondents (20%) in the probably will not get vaccinated, and 22 respondents (26%) in the definitely will not get vaccinated groups. Furthermore, 33 veterans (44%) who were not sure whether they would get vaccinated described their overall mental or emotional health as fair or poor compared with 13 veterans (33%) in the probably will get vaccinated, 30 veterans (40%) in the definitely will get vaccinated, 7 veterans (18%) in the probably will not get vaccinated, and 20 veterans (23%) in the definitely will not get vaccinated groups (Figure, B).

**Figure.**
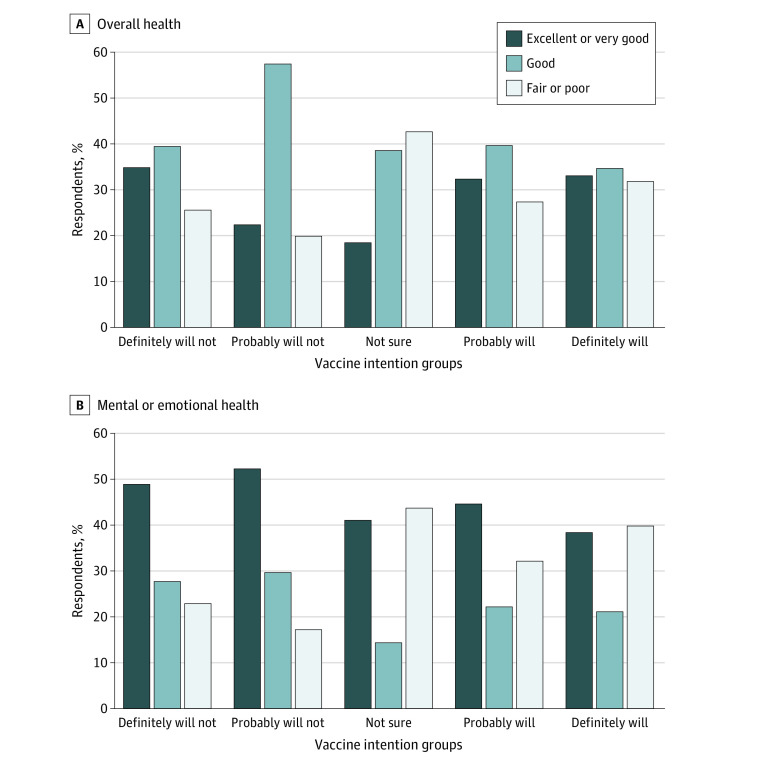
Overall Health and Mental or Emotional Health by Vaccine Intention Groups Among Veterans Who Have Not Yet Been Vaccinated

There was no significant association between vaccine intention groups and age group (χ^2^_4_ = 5.90; *P* = .21) or gender [χ^2^_2_ = 3.99; *P* = .14). However, there was a statistically significant difference *(t* = 10.9; *df* = 485; *P* < .001) between the age of those who reported being vaccinated (mean [SD], 68.9 [8.9] years) and those who reported not being vaccinated (mean [SD], 61.3 [10.9] years). Thus, older veterans were more inclined to get vaccinated than those who were younger. Associations with race and ethnicity could not be assessed owing to the relatively small numbers of American Indian or Alaska Native, Asian, Black or African American, multiracial, or Native Hawaiian or other Pacific Islander (44 respondents) and Hispanic (20 respondents) respondents who were not vaccinated.

### Reasons Given for Why Veterans Were in Favor of Getting the COVID-19 Vaccination

The top motivating reason given for getting vaccinated among those who already received a COVID-19 vaccine was that “It was the best way to prevent me from getting sick from COVID-19” (462 respondents [57%]) (Table 3). This was followed by 453 respondents (56%) reporting “It will contribute to ending the COVID-19 pandemic” and 406 respondents (50%) citing “It will help life get back to the way it was before the COVID-19 pandemic.” Similar reasons in favor of getting the COVID-19 vaccination were noted among those who were not yet vaccinated but intended to get vaccinated.

**Table 3.** Reasons in Favor of Getting the COVID-19 Vaccination Rated as Highly Important in the Already Vaccinated and Definitely Will Get COVID-19 Vaccine Groups

Reason^a^	No. (%)	
	Already (n = 807)^b^	Definitely will (n = 76)^c^
It is the best way to prevent me from getting sick from COVID-19.	462 (57)	42 (55)
It will contribute to ending the COVID-19 pandemic.	453 (56)	44 (58)
It will help life get back to the way it was before the COVID-19 pandemic.	406 (50)	36 (47)
It is the best way to prevent others from getting COVID-19.	390 (48)	44 (58)
I have a health condition that makes me more at risk from COVID-19.	323 (40)	23 (30)
Drug companies were careful to ensure the safety of their COVID-19 vaccines.	311 (39)	28 (37)
My health care provider recommended it.	257 (32)	27 (36)
The VA or military recommends getting it.	225 (28)	22 (29)
I am required to get it.	64 (8)	7 (9)
People may think less of me if I don’t get it.	22 (3)	1 (1)

Abbreviation: VA, Department of Veterans Affairs.

^a^ Respondents were provided the list of 10 reasons and asked to rate each as not important, slightly important, important, very important, or highly important and were also provided an option to provide their own reasons.

^b^ Out of a total of 817 respondents who reported being vaccinated at the time of the survey, 807 provided these reasons why.

^c^ Out of a total of 85 respondents who reported that they definitely will get vaccinated, 76 provided reasons why.

Of veterans who were not yet vaccinated but who reported that they definitely will, 44 (58%) agreed that “It will contribute to ending the COVID-19 pandemic,” and 44 (58%) agreed that “It is the best way to prevent others from getting COVID-19” as the 2 topmost motivating reasons for getting the COVID-19 vaccination. The next top reason was “It is the best way to prevent me from getting sick from COVID-19” (42 respondents [55%]) (Table 3).

### Veterans’ Trusted Sources of Information and Preferred Modes of Communication About COVID-19 Vaccines

Among those in the definitely not and probably not vaccination intention groups, the most trusted sources of information included news on the radio, television, or online (32 respondents [31%]), the VA (20 respondents [19%]), Centers for Disease Control and Prevention (20 respondents [19%]), and their coworkers or classmates (20 respondents [19%]) (Table 4). Among 69 veterans who reported being unsure about vaccination, their VA health care practitioner was a top trusted source of information, compared with those who reported that they would definitely not or probably not get vaccinated (18 veterans [26%] vs 15 veterans [15%]). In these definitely not and probably not intention groups, 34 veterans (62%) cited in-depth written material about COVID-19 research as the topmost preferred mode of communication for COVID-19 vaccines, followed by talking to someone they know (13 respondents [24%]) (Table 4).

**Table 4.** Most Trusted Sources of Information and Preferred Modes of Communication About COVID-19 Among Definitely Not, Probably Not, and Unsure Vaccine Intention Groups

Response	No. (%)^a^	
	Definitely or probably not	Unsure
Most trusted sources of information^b^		
Media sources^c^	32 (31)	25 (36)
The VA	20 (19)	22 (32)
The Centers for Disease Control and Prevention	20 (19)	17 (25)
Coworkers or classmates^d^	20 (19)	5 (7)
State government	18 (18)	11 (16)
Local government	15 (15)	7 (10)
A VA health care practitioner	15 (15)	18 (26)
My contacts on social media	13 (13)	4 (6)
The National Institutes of Health	12 (12)	6 (9)
Veteran groups or organizations	10 (10)	5 (7)
Local community organizations	6 (6)	2 (3)
A non-VA health care practitioner	6 (6)	13 (19)
The US Coronavirus Task Force	4 (4)	1 (1)
My faith leader	4 (4)	3 (4)
Preferred modes of communication^e^		
In-depth written materials about COVID-19 research (print or online)	34 (62)	29 (49)
By talking to someone they know	13 (24)	14 (24)
Hearing or reading about people like them	12 (22)	7 (12)
In-depth written material from a news source (print or online)	10 (18)	11 (19)
Listening to information on the news	10 (18)	4 (7)
Written material that is directly emailed to them	9 (16)	12 (20)
Brief written material on a web site	9 (16)	15 (25)
Public Service Announcements on television or radio	7 (13)	4 (7)
Brief written material, such as a flyer or informational sheet	2 (4)	9 (15)

Abbreviation: VA, Department of Veterans Affairs.

^a^ Values indicate percentage rating as 1 of the respondents’ 3 most trusted sources of information and 3 most preferred modes of communication about COVID-19.

^b^ Includes 103 respondents in the definitely not or probably not groups and 69 respondents in the unsure group.

^c^ Includes news on the radio, online, or in newspapers.

^d^ Includes people respondent goes to work or school with or other people they know.

^e^ Includes 55 respondents in the definitely not or probably not groups and 59 respondents in the unsure group.

### Association Between Veterans’ Experiences With COVID-19 and Vaccine Intentions

To examine the association between experience with COVID-19 and vaccine intention groups, we identified 3 exposure groups among those who had not yet been vaccinated: those who had not had COVID-19 themselves nor knew anyone who had (81 veterans [24%]), those who had not had COVID-19 themselves but did know of others who had (184 veterans [54%]), and those who reported having had COVID-19 themselves (43 veterans [13%]). Those who were not sure whether they had had COVID-19 themselves (31 veterans [9%]) were excluded from this analysis.

The association between COVID-19 exposure and vaccination intentions among the 3 intention groups was statistically significant (χ^2^_4_ = 10.28, *P* = .04). Specifically, those in the no exposure group were more likely to report that they did not intend to get vaccinated: 40 respondents (49%) in the no exposure group said they did not intend to get vaccinated, compared with 63 respondents (34%) who knew someone who had COVID-19 but had not had it themselves and 20 respondents who said they had had COVID-19 and reported they did not intend to get vaccination. Those who had not had COVID-19 themselves but did know of others who had gotten sick were more likely to report that they intended to get vaccinated (83 respondents [45%]) than those who had no COVID-19 exposure (24 respondents [30%]) and those who had had COVID-19 (11 respondents [26%]). Finally, those who had had COVID-19 themselves were more likely to report that they were leaning against getting vaccinated or were not sure. Among the group who had COVID-19, 20 respondents (47%) said they definitely will not or probably will not get vaccinated, compared with 12 respondents (28%) who said they were not sure and 11 respondents (26%) who said they definitely will or probably will get vaccinated.

## Discussion

This cross-sectional survey study of veteran users of VHA health care on their attitudes toward and intentions to receive a COVID-19 vaccine describes the many reasons why vaccine hesitancy is present during the COVID-19 pandemic. Veterans who reported that they were unsure of whether or not they intended to get vaccinated also were more likely to report fair or poor overall and mental and emotional health. This suggests that viewing one’s own health as fragile may contribute to heightened concerns about COVID-19 vaccine safety and affect decisions to become vaccinated. This finding is in contrast to a study by Fisher et al^8^ in which self-reported health was not associated with vaccine hesitancy. Thus, health care practitioners should seek out veterans known to be in poor health to engage them in conversations aimed at increasing vaccine acceptance. Furthermore, veterans who had not been exposed to COVID-19 were more likely than would be expected by chance to report that they definitely will not or probably will not get vaccinated. Those who had not had COVID-19 themselves but did know of others who had gotten sick were more likely than would be expected by chance to report that they definitely would or probably would get vaccinated. Moreover, veterans who had had COVID-19 themselves were more likely than would be expected by chance to report that they were leaning against getting vaccinated (either definitely or probably) or were not sure. This may reflect the belief that having had COVID-19 conferred sufficient immunity as to make vaccination a risk not worth taking. Our finding of a significant association between COVID-19 exposure and vaccination status is in contrast to previous studies, which have reported inconclusive associations between personal COVID-19 experience and vaccine hesitancy.^7,30^

We found no significant association of vaccine intentions with age or gender among our veteran respondents, although we did find that older veterans were more inclined to get vaccinated than those who were younger, which is more in line with other cross-sectional surveys on COVID-19 vaccines.^31,32^ This suggests that VHA health care practitioners should take age into account during conversations about vaccines with veterans.

Importantly, 71% of veterans at the time of this survey reported having already received 1 or 2 doses of a COVID-19 vaccine. Thus, vaccine acceptance in this group is comparable with others who have been surveyed (57%-75% acceptance).^33^ However, vaccine hesitancy rates captured in other surveys on US adults are estimates based on stated intentions regarding vaccination and not based on those who have already been vaccinated, as in our study. In our approach, we examined reasons that had motivated those who had already gotten vaccinated to do so. Preventing COVID-19 infection to oneself and contributing to the end of the pandemic were cited as the 2 most important reasons for getting vaccinated. This is in line with VA’s communication and education efforts promoting vaccination as a way to reduce the risk of contracting COVID-19,^34^ including recent legislation to increase the reach of vaccinations among veterans not enrolled in VHA health care, their spouses, and caregivers.^35^

We also found that vaccine hesitancy is multifaceted and may be conceptualized as deliberation, dissent, distrust, and skepticism, with no 1 category responsible for decisions to receive or not receive a COVID-19 vaccine. Concern about the adverse effects from the COVID-19 vaccine (vaccine skepticism) was the most frequently cited reason for not getting vaccinated. This suggests that easy access to medical advice about potential adverse reactions needs to be provided to build trust among those who express these fears.^36^ Fear of adverse effects from COVID-19 vaccines has been reported as a common reason for vaccine hesitation or refusal in prior studies.^7,8^ Furthermore, our finding of 36% vaccine skepticism in the veteran population is comparable to the 33% of vaccine hesitancy or resistance noted in the general US adult population.^37^ Other frequently cited reasons for not getting vaccinated ranged from the newness of the COVID-19 vaccine (vaccine deliberation), preferences to gain natural immunity and use as few medicines as possible (vaccine skepticism), to lack of trust in the health care system (vaccine distrust) and vaccines in general (vaccine dissent), which have also been noted previously.^7^ Numerous studies^38,39,40^ and a call from the US Surgeon General^41^ have sought to address these concerns by advocating for one-to-one conversations between those who are vaccine hesitant and trusted others, whether that be a health care practitioner or a peer from their social network. For veterans, we found that the VA system, the Centers for Disease Control and Prevention, coworkers or classmates, and VHA health care practitioners were trusted sources of information, but who was considered most trusted depended on whether an individual reported they would definitely not or probably not get vaccinated, or were unsure about vaccination. This points to the importance of creating multifaceted, specific messaging that could be used by VHA health care practitioners to address different types of hesitancy through involving those most trusted by veterans.^42^ Receiving information about COVID-19 vaccines from coworkers in a non-VA study was shown to be associated with reduced perceived efficacy of COVID-19 vaccines.^43^ VA employees sharing information with colleagues need as much up-to-date information on vaccines as possible.

### Limitations

This study has some limitations. Our survey is limited by capturing attitudes toward and intentions to receive a COVID-19 vaccine at only 1 point in time. However, this is the first survey of veterans of which we are aware that is focused on COVID-19 vaccination, and it represents the most up-to-date knowledge we have about why some veterans are hesitant to receive a COVID-19 vaccine. Additionally, our survey attained a response rate of 34%. Although this is not high, it is similar to what has been reported from prior studies on the VIP.^18^ Although the survey included an open-ended question for capturing additional reasons for not receiving and in favor of receiving a COVID-19 vaccine, these data were not included in the current analyses. As with any self-reported data, response bias might have impacted the validity of the results of this survey study as well.^44^ Another limitation is that the COVID-19 exposure measure used in our study did not account for several direct and indirect COVID-19 severity measures, including duration of symptoms, time elapsed since infection, COVID-19–related deaths of close friends or family, and the way in which the pandemic affected respondents (eg, financially, mental well-being). Additionally, some potential demographic correlates of vaccine hesitancy, including race and ethnicity, could not be reliably assessed owing to small numbers of survey respondents in the relevant groups.

## Conclusions

In this survey study, we found that fears about adverse effects and worry about the newness of vaccines were the primary reasons veterans reported for not getting vaccinated, reflecting vaccine skepticism and deliberation. Reasons for vaccine hesitancy identified in this study may be addressed through conversations with VA coworkers and VHA health care practitioners, trusted sources of information for veterans, to emphasize societal and community benefits of vaccines, and benefit to one’s health, especially among veterans who do not report good health.

## Notes

Supplement. **eAppendix.** Survey Text
