# Error in Abstract

**DOI:** 10.1001/jamanetworkopen.2021.41483

**Published:** 2021-12-06

In the Original Investigation titled “Attitudes and Intentions of US Veterans Regarding COVID-19 Vaccination,”^1^ published November 3, 2021, there was an error in the number and percentage of transgender or nonbinary individuals in the Abstract. This should have appeared as 11 (<1%). This article has been corrected.^1^
