# Supplementary material for: Attitudes and Intentions of US Veterans Regarding COVID-19 Vaccination
Source: JAMA Netw Open. 2021 Nov 3;4(11):e2132548. doi: 10.1001/jamanetworkopen.2021.32548 (PMC8567110; doi:10.1001/jamanetworkopen.2021.32548)
Supplement: Supplement. — eAppendix. Survey Text [file jamanetwopen-e2132548-s001.pdf]

Supplemental Online Content

Jasuja GK, Meterko M, Bradshaw LD, et al. Attitudes and intentions of US veterans regarding COVID-19 vaccination. *JAMA Netw Open*. 2021;4(11):e2132548. doi:10.1001/jamanetworkopen.2021.32548

eAppendix. Survey Text

This supplemental material has been provided by the authors to give readers additional information about their work.

© 2021 Jasuja GK et al. *JAMA Network Open.*

eAppendix. Survey Text

The VA would like to better understand Veteran attitudes toward the current Coronavirus pandemic and COVID-19 vaccination. Your answers to the questions in this survey will allow VA to provide more helpful information to Veterans and better serve their healthcare needs.

Your answers will be combined with those from many other Veterans whenever the results of this survey are reported. Your responses will never be reported in a way that makes it possible to identify you individually.

Your participation is completely voluntary. If you decide not to complete the survey, that will have no effect on the health care you receive, your eligibility for VA benefits, or your access to the COVID-19 vaccine.

We hope you will choose to take the survey. Thank you in advance for helping VA ensure the health and safety of all Veterans who receive care at the VA.

About COVID-19 and the Pandemic

1) Do you currently have or have you had COVID-19?
   a) Yes (Skip to Q2)
   b) No (Skip to Q3)
   c) Not sure (Skip to Q3)

2) How sick from COVID-19 are you or were you?
   a) Not sick at all (Skip to Q5)
   b) Mildly sick (Skip to Q5)
   c) Moderately sick (Skip to Q5)
   d) Very sick (Skip to Q5)

3) In your opinion, how likely is it that you will get COVID-19?
   a) Definitely will not (Skip to Q5)
   b) Probably will not (Skip to Q4)
   c) Not sure (Skip to Q4)
   d) Probably will (Skip to Q4)
   e) Definitely will (Skip to Q4)

4) If you were to get COVID-19, how sick do you think you would be?
   a) Not at all sick
   b) A little bit sick
   c) Moderately sick
   d) Very sick

5) Do you know anyone else who currently has or has had COVID-19? Check all that apply.
   a) Yes, a family member
   b) Yes, a friend
   c) Yes, a neighbor
   d) Yes, a coworker
   e) Yes, some other person
   f) I do not know anyone who currently has or has had COVID-19

© 2021 Jasuja GK et al. *JAMA Network Open.*

6) How strongly do you agree or disagree with each of the following statements about COVID-19?

Grid format; strongly disagree to strongly agree for each item in list; 5-point scale with "Neither agree nor disagree" mid-point. In all grids, order of presentation will be randomized across respondents.

a) COVID-19 is a hoax
b) COVID-19 is manmade
c) People are **not** being told the truth about how well the COVID-19 vaccines **work**
d) People are **not** being told the truth about the **safety** of the COVID-19 vaccines

7) Which of these sources, if any, do you use to get information from regarding COVID-19?
First presentation: All options listed; randomize order of presentation across respondents; instruction: Please select all that apply.

a) My VA health care provider
b) My non-VA health care provider
c) My faith leader
d) People I go to work or school with or other people I know
e) My contacts on social media
f) News on the radio, TV, online, or in the newspapers
g) The U.S. Coronavirus Task Force
h) The Centers for Disease Control and Prevention (CDC)
i) The National Institutes of Health (NIH)
j) The VA
k) State government
l) Local government
m) Veterans groups or organizations (Please specify which Veteran service group(s) or organization(s): ___________________)
n) Local community organizations
o) None of the above (note: single select only)

Second presentation: <u>If 4 or more selected</u>, display the subset of items that were selected in the same order they were first presented. Instruction:

Of the sources you selected, which are the ones that you **trust the most** to provide correct information about COVID-19? You may select up to 3 as your most trusted.

If 3 or fewer sources selected from initial presentations, then no second presentation. If "none of the above" selected, then no second presentation.

© 2021 Jasuja GK et al. *JAMA Network Open.*

8) In addition to those we just asked about, are there other sources you use to get information about COVID-19?
   - ☐ Yes, I would like to add one or more additional sources (Continued to Q9)
   - ☐ No other sources to add at this time (Skip to Q15)

9) Please use the space below to tell us about **one of the additional sources** of information about COVID-19 that you use.

10) Is this additional source of information about COVID-19 one of those that you **trust the most**?
   - ☐ Yes
   - ☐ No
   - ☐ Not sure

11) Are there any more additional sources you use to get information about COVID-19 that you want to tell us about?
   - ☐ Yes (Continue to Q6E)
   - ☐ No (Skip to Q7)

12) Please use the space below to tell us about **one last** additional source of information about COVID-19 that you use.

13) Is this additional source of information about COVID-19 one of those that you **trust the most**?
   - ☐ Yes (Q7 next)
   - ☐ No (Q7 next)
   - ☐ Not sure (Q7 next)

© 2021 Jasuja GK et al. *JAMA Network Open.*

COVID-19 Vaccines

The next questions are about vaccinations in general and COVID-19 vaccines in particular.

14) In the past 5 years, how often have you received the annual flu shot?
    p) Never/Sometimes/Usually/Always *(Note: this is SHEP/CAHPS NSUA response scale)*

15) Have you received a COVID-19 vaccine?
    a) Yes, the single dose kind (Skip to Q17)
    b) Yes, the first shot of two (Skip to Q16)
    c) Yes, the second shot of two (Skip to Q17)
    d) No (Continue to Q18)

16) Will you be going back for your second shot?
    a) Yes
    b) No
    c) Not sure

17) Where did you get your COVID19 vaccination?
    - ☐ At a VA facility
    - ☐ Outside the VA

18) Do you intend to get a vaccine for COVID-19?
    a) Definitely will not (Skip to Q21)
    b) Probably will not (Skip to Q19)
    c) Not sure (Skip to Q19)
    d) Probably will (Skip to Q20)
    e) Definitely will (Skip to Q20)

19) If you were to decide to get it, where would you be most likely to get your COVID-19 vaccine?
    - ☐ At a VA facility
    - ☐ Outside the VA
    - ☐ Wherever I can
    - ☐ Not sure

20) Where do you plan to get your COVID-19 vaccine?
    - ☐ At a VA facility
    - ☐ Outside the VA
    - ☐ Wherever I can
    - ☐ Not sure

© 2021 Jasuja GK et al. *JAMA Network Open.*

How strongly do you agree or disagree with the following statements about COVID-19 vaccines?

Note: Randomize order of presentation of Q21 thru Q25.

21) The vaccines available for COVID-19 are **safe**.
    a) Strongly disagree
    b) Disagree
    c) Neither agree nor disagree
    d) Agree
    e) Strongly agree

22) The vaccines available for COVID-19 are **effective**.
    a) Strongly disagree
    b) Disagree
    c) Neither agree nor disagree
    d) Agree
    e) Strongly agree

23) The vaccines available for COVID-19 are **easy to get**.
    a) Strongly disagree
    b) Disagree
    c) Neither agree nor disagree
    d) Agree
    e) Strongly agree

24) The vaccines available for COVID-19 are **necessary to help end the pandemic.**
    a) Strongly disagree
    b) Disagree
    c) Neither agree nor disagree
    d) Agree
    e) Strongly agree

25) The **VA's** COVID-19 vaccine **distribution** process is **safe**.
    a) Strongly disagree
    b) Disagree
    c) Neither agree nor disagree
    d) Agree
    e) Strongly agree

Those who answered A, B, or C to Q15: Skip to Q30

© 2021 Jasuja GK et al. *JAMA Network Open.*

26. (First grid question.) Which of the following reasons, if any, explain why you have not received a COVID-19 vaccine at this time? Please indicate how important each reason is for you.

(Second grid question.) Which of these **additional reasons**, if any, explain why you have not received a COVID-19 vaccine at this time? Please indicate how important each reason is for you.

Place items 1-9 in the first grid, and items 10-18 in the second grid. Within each grid, order of presentation will be randomized across respondents. Response options: Not Important / Slightly Important / Important / Very Important / Highly Important.

1. I am concerned about side effects from the vaccine.
2. I am concerned about vaccine safety related to pregnancy and/or breastfeeding.
3. You need to get 2 shots about 1 month apart for the vaccine to work.
4. I don't have transportation.
5. I don't know how to get a COVID-19 vaccine.
6. I am eligible for a COVID-19 vaccine but have not yet been able to get an appointment.
7. I am allergic to vaccines.
8. I do not like needles.
9. It is against my religious or philosophical beliefs.
10. I prefer gaining natural immunity.
11. I prefer to use as few medicines as possible.
12. I do not trust vaccines.
13. I'm worried it will alter my DNA.
14. The COVID vaccine is new, so I want to wait a while before deciding.
15. Getting a COVID-19 vaccine is too difficult.
16. I do not trust the healthcare system to act in my best interests.
17. I've already had COVID-19 so I don't believe that I need a COVID-19 vaccine.
18. I am not eligible for a COVID-19 vaccine at this time.

27. In addition to the reasons listed above, is there another reason that you have not received a COVID-19 vaccine that is important to you?

- ☐ Yes, there is another reason I would like to add (Continue to Q28)
- ☐ I have no other reason to add at this time (Skip to Q30)

28. Please use the space below to tell us about that reason.

29. How important is this as a reason why you have not received a COVID-19 vaccine at this time?
    - ☐ Slightly Important
    - ☐ Important
    - ☐ Very Important
    - ☐ Highly Important

30. Here are some reasons that people give **in favor of** getting a vaccine for COVID-19. Please indicate how important each reason is for you.

Grid format; response options: Not Important / Slightly Important / Important / Very Important / Highly Important

© 2021 Jasuja GK et al. *JAMA Network Open.*

1. It's the best way to prevent me from getting sick from COVID-19.<br>2. It's the best way to prevent others from getting COVID-19.<br>3. I have a health condition that makes me more at risk from COVID-19.<br>4. The VA or Military recommends getting it.<br>5. People may think less of me if I don't get it.<br>6. I am required to get it.<br>7. My healthcare provider recommended it.<br>8. It will contribute to ending the COVID-19 pandemic.<br>9. It will help life get back to the way it was before the COVID-19 pandemic.<br>10. Drug companies were careful to ensure the safety of their COVID-19 vaccines.

31. In addition to the reasons listed above, is there another reason **in favor of** getting a vaccine for COVID-19 that is important for you?

- ☐ Yes, there is another reason I would like to add (Continue to 32)
- ☐ I have no other reason to add at this time (Skip to Q34)

32. Please use the space below to tell us about that additional reason.

33. How important for you is this additional reason **in favor of** getting a vaccine for COVID-19?

- ☐ Slightly Important
- ☐ Important
- ☐ Very Important
- ☐ Highly Important

34. How would you prefer to learn more about the COVID-19 vaccines?

The list will be presented twice.

(1) first presentation – full list, instruction: Please check all that apply. Order of presentation of options will be randomized across respondents.

(2) Second presentation – only the subset of previously selected items, presented in same order as in first presentation. Second presentation instruction:

Of those that you selected, which are the ones that you **prefer the most** as ways to learn more about COVID-19 vaccines? You may select up to 3 as your most preferred.

If 3 or fewer methods selected from initial presentations, then no second presentation.

a. Brief written material such as a flyer or information sheet

b. Brief written material on a web site

c. Hearing or reading about people like me

d. Written material that is e-mailed directly to me

© 2021 Jasuja GK et al. *JAMA Network Open.*

e. Listening to information on the news

f. In-depth written material from a news source (print or online)

g. In-depth written material about CoVID-19 vaccine research (print or online)

h. Public service announcements (PSAs) on television or radio

i. By talking to someone I know

j. None of the above

35. In addition to those we just asked about, are there **other ways of learning more about COVID-19 vaccines** that you would also prefer to have available?

- ☐ Yes, I would like to add one or more additional ways (Continued to Q36)
- ☐ No other way to add at this time (Skip to Q41)

36. Please use the space below to tell us about **one of the additional ways** of learning more about COVID-19 vaccines that you would prefer to have available.

37. Is this additional way of learning more about COVID-19 vaccines **one of the ways that you prefer the most**?

- ☐ Yes
- ☐ No
- ☐ Not sure

38. Are there any more additional ways of learning more about COVID-19 vaccines that you want to tell us about?

- ☐ Yes (Continue to Q39)
- ☐ No (Skip to Q41)

39. Please use the space below to tell us about **one last additional way** of learning more about COVID-19 vaccines that you would prefer to have available.

40. Is this additional way of learning more about COVID-19 vaccines **one of the ways that you prefer the most**?

- ☐ Yes
- ☐ No
- ☐ Not sure

41. Is there anything else you think VA should know about your views of the COVID-19 vaccines?

- ☐ I have no additional comments at this time. (Skip to Q43)
- ☐ Yes, I do have some additional comments. (Continue to 42)

42. Please use this space to tell us your additional thoughts about COVID-19 vaccines.

© 202

About Your Health Care

The next questions ask about your healthcare overall, both in the VA and in general.

43. A primary care provider (PCP) is the healthcare provider you usually see if you need a check-up, want advice about a health problem, or get sick or hurt. Which of the following describes your PCP?
    - A VA provider that I see at a VA facility
    - A non-VA provider in the community paid for by VA
    - A non-VA provider in the community not paid for by VA
    - I don't currently have a PCP (Skip to Q48)

44. How strongly do you agree or disagree with each of the following statements about your **relationship with your primary care provider**? *(Grid; 5-point agree/disagree scale)*
    - I can tell my primary care provider anything, even things that I might not tell anyone else
    - My primary care provider sometimes pretends to know things when he/she is not really sure
    - I completely trust my primary care provider's judgment about my medical care
    - My primary care provider cares more about cutting down costs than about doing what is needed for my health
    - My primary care provider would always tell me the truth about my health, even if there was bad news
    - My primary care provider cares as much as I do about my health
    - If a mistake was made in my treatment, my primary care provider would try to hide it from me

45. Which of the following best describes how you and your primary care provider usually go about **making decisions about your care**?
    - My primary care provider generally makes decisions for me
    - I like to know the options available but still let my primary care provider decide for me
    - My primary care provider and I make decisions together
    - I make decisions for myself, after considering the advice of my primary care provider
    - I always make my own decisions, independently of the advice of my primary care provider

46. How long have you been using health care services provided by the VA?
    - Less than 1 year
    - 1 to 4 years
    - 5 to 9 years
    - 10 or more years

47. In the past 6 months, did you get health care **from the VA**? Please select all that apply. *(Note: multiple "yes" options can be checked; if "no" selected, that will be the only option permitted)*.
    - Yes, I got health care at a VA facility
    - Yes, I got health care in the community paid for by VA
    - No VA health care in the last 6 months

© 2021 Jasuja GK et al. *JAMA Network Open.*

48. In the last 6 months, about how often did you get health care **anywhere**?
- ☐ No health care in the last 6 months
- ☐ Less than once a month
- ☐ About once a month
- ☐ About 2-3 times every month
- ☐ About once a week
- ☐ More than once a week

49. How strongly do you agree or disagree with the following statements about **VA health care?** *(Grid; 5-point agree/disagree response scale)*
- ☐ I trust the VA to put my medical needs above all other things.
- ☐ The medical skills of the VA doctors and nurses are NOT as good as they should be.
- ☐ I trust the VA to give me the information I need about my treatment.
- ☐ The VA will NOT give me the best possible care.

© 2021 Jasuja GK et al. *JAMA Network Open.*

About You

Finally, we would appreciate knowing a little more about you so that we can understand how well the survey has captured the opinions of all different groups of Veterans. The results of this survey will never be reported in a way that would permit individual respondents to be identified.

50. In general, how would you rate your overall health?
- ☐ Excellent
- ☐ Very Good
- ☐ Good
- ☐ Fair
- ☐ Poor

51. In general, how would you rate your overall mental or emotional health?
- ☐ Excellent
- ☐ Very Good
- ☐ Good
- ☐ Fair
- ☐ Poor

52. Which of the following conditions do you have? Please check all that apply.

- ☐ Cancer
- ☐ Chronic kidney disease
- ☐ Chronic obstructive pulmonary disease (COPD)
- ☐ Asthma (moderate-to-severe)
- ☐ Cystic fibrosis
- ☐ Pulmonary fibrosis (damaged or scarred lungs)
- ☐ Heart conditions, such as heart failure, coronary artery disease, or cardiomyopathies
- ☐ Hypertension or high blood pressure
- ☐ Stroke or blocked blood vessels or blood supply to the brain
- ☐ Neurologic conditions, such as dementia
- ☐ Down Syndrome
- ☐ Obesity
- ☐ Liver disease
- ☐ Pregnancy
- ☐ Sickle cell disease or Thalassemia (a type of blood disorder)
- ☐ Smoking
- ☐ Type 2 or Type 1 diabetes mellitus
- ☐ Weakened immune system from blood or bone marrow transplant, immune deficiency, HIV/AIDS, organ transplant, or use of immune-weakening medicines
- ☐ None of the above

© 2021 Jasuja GK et al. *JAMA Network Open.*

53. What is the highest grade or level of school that you have completed?
- ☐ 8th grade or less
- ☐ Some high school, but did not graduate
- ☐ High school graduate or GED
- ☐ Some college or 2-year degree
- ☐ 4-year college graduate
- ☐ More than 4-year college degree

54. Are you of Hispanic or Latino origin or descent?
- ☐ Yes, Hispanic or Latino
- ☐ No, Not Hispanic or Latino

55. What is your race? Mark one or more.
- ☐ White
- ☐ Black or African-American
- ☐ Asian
- ☐ Native Hawaiian or other Pacific Islander
- ☐ American Indian or Alaska Native

56. What is your gender?
- ☐ Man
- ☐ Woman
- ☐ Transgender Man
- ☐ Transgender Woman
- ☐ Non-binary
- ☐ Other

57. Do you consider yourself to be:
- ☐ Heterosexual or straight
- ☐ Gay
- ☐ Lesbian
- ☐ Bisexual
- ☐ Other
- ☐ I am not sure

58. During the past 6 months, how often were you **unable to pay** your bills for any basics such as food, housing, heating or health care?
- ☐ Never
- ☐ Rarely
- ☐ Sometimes
- ☐ Often
- ☐ Always

Thank you for taking the time to help VA provide better care to you and to all Veterans!

© 2021 Jasuja GK et al. *JAMA Network Open.*
